# Photomobile Polymer–Piezoelectric Composite for Enhanced Actuation and Energy Generation

**DOI:** 10.1021/acsaom.3c00227

**Published:** 2023-09-28

**Authors:** Domenico Sagnelli, Amalia D’Avino, Massimo Rippa, Ambra Vestri, Valentina Marchesano, Giuseppe Nenna, Fulvia Villani, Gustavo Ardila, Sonia Centi, Fulvio Ratto, Lucia Petti

**Affiliations:** † Institute of Applied Sciences and Intelligent Systems of CNR, Pozzuoli 80072, Italy; ‡ Energy and Sustainable Economic Development, ENEA, Italian National Agency for New Technologies, Portici Research Centre, Portici, Naples 80055, Italy; § CNRS, Grenoble INP, IMEP-LaHC, Univ. Grenoble Alpes, Univ. Savoie Mont Blanc, Grenoble F-38000, France; ∥ Nello Carrara Institute of Applied Physics of CNR, Sesto Fiorentino 50019, Italy

**Keywords:** PMP, azobenzene, noble-metal nanoparticles, hybrid materials, smart materials, solar energy harvesting

## Abstract

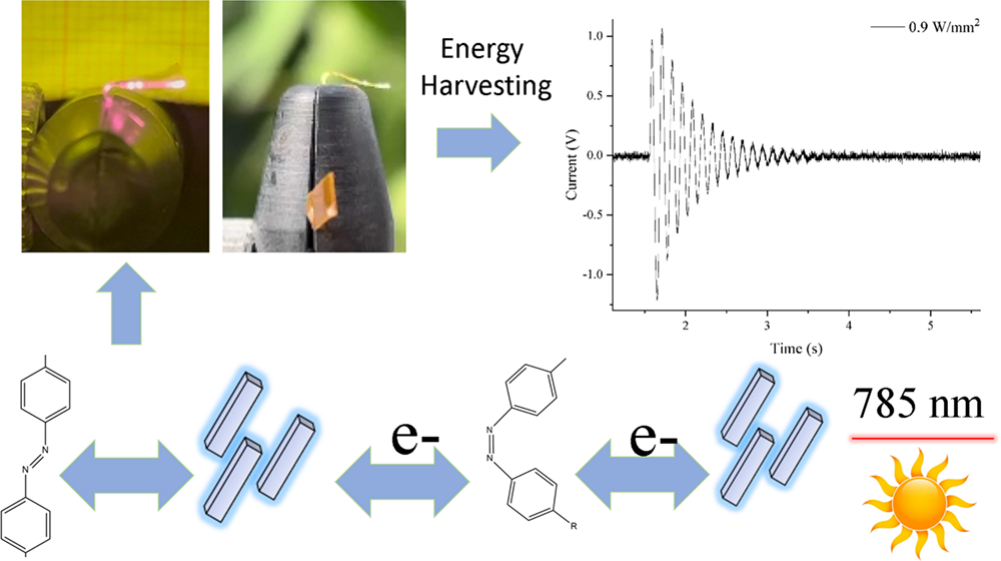

In this study, we present an innovative approach to increase the quantum yield and wavelength sensitivity of photomobile polymer (PMP) films based on azobenzene by doping the polymer matrix with noble metal nanoparticles. These doped PMP films showed faster and more significant bending under both UV as well as visible and near-infrared light regardless of whether it was coherent, incoherent, polarized, or unpolarized irradiation, expanding the potential of PMP-based actuators. To illustrate their practical implications, we created a proof-of-concept model of power generation by coupling it to flexible piezoelectric materials under simulated sunlight. This model has been tested under real operating conditions, thus demonstrating the possibility of generating electricity with variable light exposure. Additionally, our synthetic protocol is solvent-free, which is another benefit of environmental relevance. Our research lays the groundwork for the development of sunlight-sensitive devices, such as photomechanical actuators and advanced photovoltaic modules, which may break ground in the thriving field of smart materials. We are confident that the presented findings will contribute to the ongoing discourse in the field and inspire additional advances in renewable energy applications.

## Introduction

Smart materials, with their unique reactive properties, have captivated both the scientific community and the industrial sector due to their wealth of potential applications. Among them, smart material-based actuators have made great strides, finding their place in different areas such as sensors, motors, soft robotics, oscillators, and optical devices. These devices owe their functionality to the reversible deformation exhibited by the underlying materials in response to external stimuli such as light, humidity, pH, heat, and electric fields.^1−9^ The integration of these materials into multifunctional architectures promises groundbreaking scenarios. In particular, within the Future and Emerging Technologies initiative of the EU Horizon2020 work program, the project entitled “Photo-Piezoactuators based on Light Sensitive Composite” aimed at an all-polymer composite made with piezoelectric (PZL) films and components capable of converting sunlight into mechanical vibrations as an ideal solution to outperform existing photovoltaic technologies in terms of cost-effectiveness, durability, lightness, and flexibility.^10^ However, while PZL polymers such as polyvynilidene fluoride (PVDF) have matured into a technology of increasing interest for harvesting ambient energy,^11,12^ there is still no sunlight-sensitive material capable of supporting vibrations. Here, we demonstrate a viable solution to take a radical step in this promising direction.

Among the plethora of smart materials, liquid crystalline polymer (LCP)-based actuators have emerged as a particularly promising route for real-world applications. There are two main families of liquid crystals (LCs): thermotropic LCs, which undergo a change of state in response to heat, and lyotropic LCs, which exhibit intermolecular order after dissolution in a suitable solvent (e.g., DNA). Thermotropic LCs are known for their intermolecular alignment.^13,14^ LCPs, typically derived from nematic LCs, can be easily oriented into a nematic order by temperature, mechanical means such as shear, or other physical triggers such as electromagnetic fields. Polymerized and cross-linked nematic LCs produce frozen nematic phases that respond to certain stimuli, depending on the moieties integrated into the network.^15,16^

Photomobile polymers (PMPs) are a particular class of LCPs that move in response to light and are often produced as thin films.^17−21^ The response of PMPs depends on various factors, including the choice of photosensitive moieties, the polarization of light, the power density, and the composition and orientation of the liquid crystalline domains.^17,22,23^ Azobenzene moieties are often incorporated into PMPs as the driving force of movement.^24^ Indeed, azobenzene undergoes a *trans*–*cis* photoisomerization reaction induced by polarized UV light, which leads to a macroscopic deformation of the overall films.^25−28^ Interestingly, even as little as 6% azobenzene is sufficient to induce functional degrees of strain in an LCP matrix.^29^

However, the photoisomerization of azobenzene requires a strict set of conditions, namely polarized light in a particular range of UV or at least violet wavelengths (230–460 nm^30^), thus limiting its potential for practical applications. To make this technology more attractive for energy sensing and harvesting, the formulation of PMPs as nanocomposite hybrids has become a recurring effort.^31−33^ Various nanocomposite systems have already been reported in the literature, showing improved properties. For example, anisotropic gold nanoparticles are capable of absorbing near-infrared (NIR) light and converting it into heat, thus triggering a thermoelastic deformation of the surrounding polymer.^34^ Other examples are carbon nanotubes that provide higher conductivity and efficiency,^35−37^ or titanium nitride nanoparticles that provide optical absorbance over a wide range of wavelengths between 500 and 1200 nm.^38^

In this study, we present a simple method to improve the wavelength sensitivity and photomechanical conversion efficiency of azobenzene-based PMP films, by doping with silver nanocuboids (SNCs).^39^ We hypothesize that the SNCs, designed to resonate with red light, may emit and reaccept photoelectrons to trigger the isomerization of azobenzene, thereby extending the wavelength sensitivity of the nanocomposite system to the point of continuous self-motion under sunlight. This represents the first reported use of SNCs to enhance the performance of azobenzene-based PMPs for solar energy harvesting. We believe that this approach has significant potential to accelerate the development of PMPs that can be coupled with PZL films for new PV modules.^40^

## Experimental Section

### Materials

LC monomers 4-methoxybenzoic acid 4-(6-acryloyloxyhexyloxy)phenyl ester, 4[4[6-acryloxyhex-1-yl)oxyphenyl]carboxybenzonitrile, 1,4-bis-[4-(6-acryloyloxyhexyloxy)benzoyloxy]-2-methylbenzene, and 4,4′-bis[9-(acryloyloxy)nonyloxy]azobenzene were purchased at Synthon Chemicals (Germany), and the photoinitiator bis(2,4,6-trimethylbenzoyl)-phenylphosphineoxide was from Sigma-Aldrich. Elvamide was provided by Beamco. Other chemicals were provided by Sigma-Aldrich.

### Preparation of the Cell Reactor

Photoactuator synthesis cells were prepared using glass slides and plastic spacers. The method used was preciously discussed in the literature.^41^

### Synthesis of the PMP Films

The PMP films were prepared using a mixture of LC monomers proposed by Lahikainen et al.,^29^ with some modifications. In brief, the reaction mixture was dissolved in dichloromethane and heated at 70 °C until all solvent was removed. Then, the reaction mixture was left to recrystallize at RT overnight.

**Figure 1:**
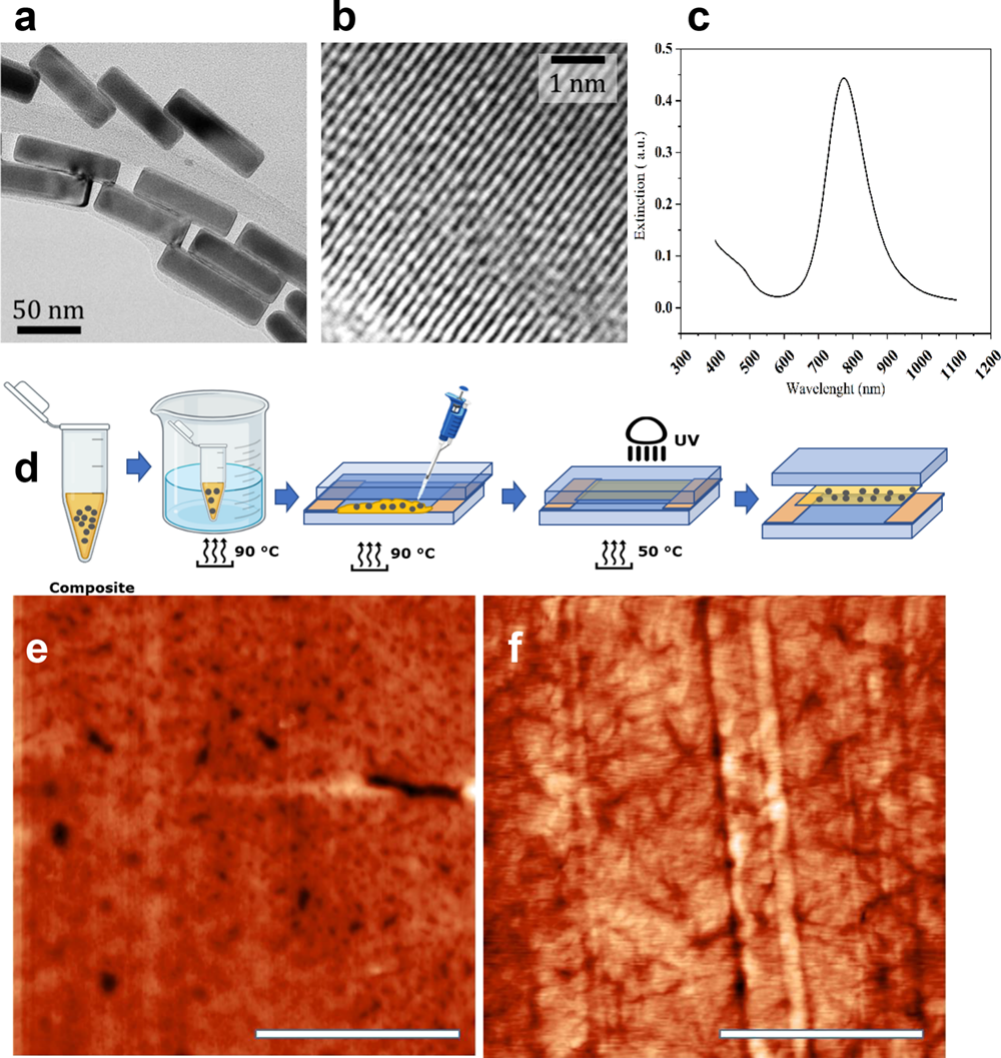
(A, B) TEM micrographs of the SNCs used in this work; (C) UV–vis–NIR spectra showing the plasmonic bands of the SNCs; (D) scheme showing the fabrication procedure for the metal-doped PMP films; and (E, F) AFM investigation of 6%Azo-PMP alone (F) or doped with SNCs (E) (scale bars are 5 μm).

The reaction cell was heated to 95 °C, and the mixture infiltrated by capillarity (Figure 1D). After the infiltration, the sample was moved on a second hot plate set at the nematic temperature (50 °C) and photopolymerized for 1 h (30 min each side) using a UV lamp emitting at 405 nm (12 mW/cm^2^) and successively left for 24 h at 50 °C.

The doping process was carried out by following a protocol described elsewhere.^41^

### Synthesis of SNCs

Silver/gold nanocuboids were prepared by overgrowing gold nanorods with a silver shell. Cetrimonium-capped gold nanorods were first synthesized according to a variant of the protocol developed by Vigderman and Zubarev^42^ that is described in detail elsewhere,^43^ in order to achieve longitudinal modes of plasmonic oscillations peaking between about 1000 and 1100 nm. Thereafter, the as-synthesized gold nanorods were coated with a silver shell according to the protocol reported in refs (39) and (44). In brief, particles were transferred at a nominal concentration of 200 μM Au in a solution containing 20 mM cetrimonium chloride (CTAC) and supplemented with 400 μM AgNO3 and 1.6 mM ascorbic acid. This molar ratio of Ag:Au around 2:1 was intended to convey a blue shift of the longitudinal modes of the gold nanorods exceeding 200 nm.^39^ After 2 h at 70 °C, particles were brought to a concentration of 1.6 mM Au in a 100 mM acetate buffer pH 5.0 containing 500 μM CTAC, 0.005% (w/w) polysorbate 20 and 50 μM mPEG-SH,^45^ in order to obtain an amphiphilic termination and delay the onset of oxidative degradation of the silver shell. After 2 h under gentle agitation at 37 °C, suspensions were purified and transferred to ultrapure water containing 0.005% (w/w) polysorbate 20 before subsequent manipulation. Likewise, silver/gold nanocubes were prepared by overgrowing gold nanospheres with a silver shell. Gold nanospheres were synthesized from the same seeds used for the preparation of the gold nanorods, which were diluted 1:80 in 73 mM CTAC, 27 mM ascorbic acid, and 180 μM HAuCl4. The protocol for the addition of silver and mPEG-SH was the same as above. However, in this case, the ratio Ag: Au was about 10:1. After PEGylation, particles were stored under the same conditions and the same total concentration of noble metal as the silver/gold nanocuboids. For integration in an aromatic polymer, all particles were formulated as a dry power by implementing a freeze-dryer^46^ from Labconco (MO, USA) and stored under low vacuum, in an effort to further hinder the oxidative loss of the silver shell.

### UV/Vis Spectral Characterization

Spectral characterization in the UV/vis range of the PMP films (thickness ≈ 50 μm) was performed using a JASCO V-650 UV/vis Spectrophotometer (accuracy 0.5 nm, range 190–850 nm, Oklahoma, OK, USA). Both total percentage transmittance *T* (%) and total percentage reflectance *R* (%) were measured using a JASCO ISN-722 integrating sphere (inside diameter 60 mm, range 200–870 nm).

The PMPs were further characterized to understand if the metal nanoparticles would reduce the decay time of the cis isomer of azobenzene.^51^ The PMPs were irradiated for 10 min using a 405 nm polarized laser at a power density of 1.5 mW/cm^2^. Thereafter, their optical absorbance was measured after 75 min in the dark. The optical absorbance was calculated as *A* (%) = 100 – *T* (%) – *R* (%).

### Thermographic Measurements

Thermographic measurements of the PMP films during and after laser irradiation were performed using an AVIO TVS 500 LWIR camera (VOx microbolometer, spectral range 8–14 μm, Focal Plane Array (FPA), 320 × 240, Noise Equivalent Temperature Difference (NETD) ∼ 0.05 K) mounted on a standard 22 mm lens. For time-resolved trends, images were recorded at a frame rate of 20 Hz. The emissivity of the film was set to 0.93. All measurements were realized at a laboratory temperature of 23 °C and humidity of 50%.

### Atomic Force Microscopy

The PMP samples were morphologically characterized by atomic force microscopy (AFM from the company NT-MDT). The analysis was performed in a semicontactless configuration using high-resolution golden silicon cantilevers.

### Polarized Optical Microscopy

An Olympus BX60 optical microscope with crossed polarizers and a magnification of 20× was used to characterize the polymer films. The images of bare and metal-doped PMPs were taken at room temperature, and the samples were set both parallel and 45 deg relative to one of the polarizers.

### Bending and Relevant Kinetic Characterization

In order to study the dynamic response of the PMPs, both bare and metal-doped actuators were cut as cantilevers (5 mm × 1 mm) and irradiated at 405, 457, 532, and 785 nm with 100:1 polarized laser. The setup was composed of a neutral density filter, a retarder waveplate (λ/2), a focusing lens, and a sample holder mounted on a 3D translator (Supplementary Figure 3).

The movements of the cantilevers were recorded at 60 fps. The bending angle was measured with respect to the initial position of the cantilever. The angle was converted in radiant as well as in arc length (mm), and the speed was calculated in m/s. The time to achieve maximum bending was derived by counting the number of frames needed for the cantilever to complete its deformation.

### PMP–PZL Proof-of-Concept Harvesting System

The PMP layer was laminated with a copper layer (≈10 μm) to ensure fast heat dissipation under light concentration. The xenon arc lamp can be controlled up to 150 W by a power supply; the experiments were conducted with an optical power range from 0.3 to 0.9 W/mm^2^. The light was chopped (1 Hz) by a Thorlabs optical chopper and was focalized by means of a lens with a 5 cm focal length. The PZL device is an RS vibration sensor (length 41 mm × width 16 mm) PVDF-based (thickness 200 μm). The PZL has been stretched by two bidhesive Kapton tapes by 5 cm in order to increase the mechanical leverage the PMP has been placed against the PZL device as shown in the Supporting Information, and the electrical measurements were performed with the use of an oscilloscope (Lecroy LC684D).

## Results and Discussion

### Synthesis and Characterization of Nanocomposite PMPs

The dopants that we tested in this work are SNCs, which we chose for their optical versatility and rich electrochemical profiles. In particular, we used PEGylated silver/gold nanocuboids that exhibit a distinct band of longitudinal plasmonic oscillations with a peak around 760 nm (Figure 1A), well separated from the optical absorbance of azobenzene. The hydrodynamic size and electrokinetic potential of the PEGylated SNCs were (68 ± 12) nm and (−17 ± 2) mV. The latter is consistent with the anionic profile observed in various types of more standard gold nanorods processed under similar conditions.^47,48^ HR-TEM micrographs of these SNCs confirmed their core/shell structure, with silver shells showing no clear signs of oxidation but high crystalline quality and regular *d* spacing between lattice planes around 200 pm (Figure 1 B,C).^49^

For easy integration into PMP systems, the SNCs were freeze-dried and mixed as a powder with the LC mixes. The composite blends were melted, injected into a homemade cell, and cured.^41,50^ The first characterization we performed on the nanocomposite hybrids concerned their birefringence to understand if the LCs maintained their nematic organization after doping. Interestingly, PMPs containing both azobenzene and SNCs (Supplementary Figure 1A,B) showed an increase in optical transmittance when the films were rotated 45° relative to the polarizers (Supplementary Figure 1). When the azobenzene was removed from the mixtures (Supporting Information, Figure 1C,D), this effect became even more evident (Supporting Information, Figure 1C,D). This result indicates that our process for nanocomposite synthesis is consistent and reproducible and that the SNCs (2, 1, 0.5, 0.25% w/w) do not disrupt the nematic order of their host. We have previously shown that adding up to 6% ZnO nanoparticles in a nematic mixture can disrupt the nematic organization of the PMPs.^41^

Subsequently, the PMP films were characterized for their topography in order to understand whether the SNCs influence their surface roughness. AFM and UV–vis–NIR spectroscopy measurements are reported in Figure 1. AFM measurements showed that the SNCs do not segregate on the surface but are well dispersed within the polymer matrix, probably due to intermolecular interactions between the LC and PEG chains used as particle coatings (Figure 1E). The PMPs without SNCs showed no holes but only the typical features of the rubbing process (Figure 1F).

### Understanding the Influence of SNCs on Azobenzene Isomerization in the PMPs

**Figure 2:**
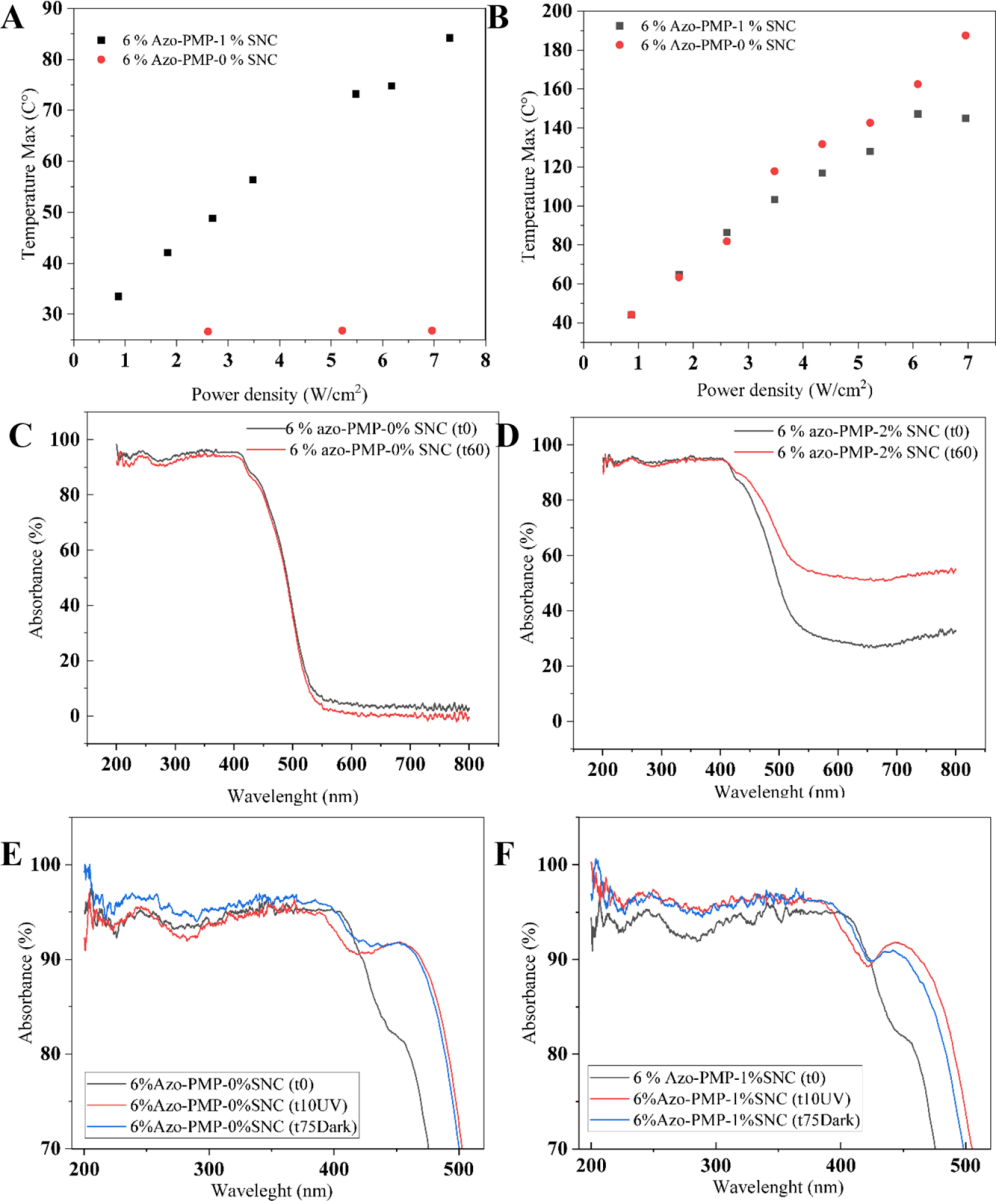
(A, B) Thermometric measurements for 6%Azo-PMP and 6%azo-PMP-SNC irradiated with polarized laser light at 785 (A) or 457 nm (B). (C, D) Absorbance pre-/postirradiation of PMPs in the case of 6%Azo-PMP-0%SNC (C) and 6%azo-PMP-2%SNC (D) irradiated with a 785 nm laser for 1 h. (E, F) Dark isomerization experiment. (E) 6%Azo-PMP showing that after 75 min in the dark, there was no *cis*-isomerization. (F) 6%Azo-PMP-1%SNC showing that 75 min in the dark was sufficient for the azobenzene to start to reisomerize into a *cis* state.

After synthesizing and characterizing the topography of the nanocomposite films, we shifted our attention toward the interactions between the SNCs and the PMPs. We conducted thermal response measurements using laser light at two different wavelengths, 457 and 785 nm, and a thermal imaging camera with the procedures described in the Materials and Methods section. The 785 nm wavelength, which does not induce isomerization of azobenzene, was tested first.^41^ It overlapped the plasmonic resonance of the SNCs (Figure 1C), leading to a heat increase proportional to the power density on the surface of the SNC-doped PMP (Figure 2A black curve).^51^ However, the SNC-free PMP did not undergo any detectable heating, in agreement with the low optical absorbance of the LC matrix in the near-infrared window (Figure 2A red curve). We then used a laser emitting at 457 nm, where both PMPs exhibited consistent heat buildup, attributable to the optical absorbance of azobenzene (Figure 2B). Notably, the SNC-doped PMP exhibited temperature saturation at high power density, suggesting a fast dissipation effect (Figure 2B black curve). Heat dissipation in nanocomposite PMPs has been previously observed in the case of ZnO nanoparticles.^41,52^

Given the well-established fact that Au- and Ag-based nanoparticles can emit photoelectrons in a vacuum when excited to their plasmonic resonance,^53,54^ we hypothesized that photoelectrons emitted from the SNCs may drive the isomerization of azobenzene.^53,55^ To verify this, we measured the optical absorbance of the 6% Azo-PMP-SNC and 0% Azo-PMP-SNC films both before and after irradiation with light at 785 nm. The azobenzene-free PMP showed no change in absorbance with irradiation (Figure 2C). Conversely, the presence of azobenzene in the polymer triggered an abrupt absorbance transition (Figure 2D), indicating a change in the chemical properties of the polymer matrix, likely due to the isomerization of azobenzene. We conjecture that this effect may originate from an exchange of electrons between the SNCs and neighboring azobenzene moieties, which may then propagate further through the polymeric backbone.^56^

To strengthen our hypothesis, we explored the potential for a catalytic influence on the dark isomerization, a process that refers to the activation of the isomerization of azobenzene from its excited cis state to the ground trans state in the absence of light.^23,46^ Previous research has suggested that gold nanoparticles can catalyze this transition.^56,57^ Accordingly, we examined the evolution of the optical absorbance of azobenzene and its *trans*–*cis*–*trans* isomerization for freshly prepared 6% Azo-PMP and 6% Azo-PMP-SNC, as reported in Figure 2E,F. Both films were irradiated with a led-UV lamp (12 mW/cm^2^) centered at 405 nm for 10 min and their absorbance was measured again (red line, Figure 2E,F). Subsequently, the PMPs were kept for 75 min in the dark and finally showed different patterns of optical absorbance. When the SNCs were present, the azobenzene component displayed a much faster change of optical absorbance (blue line, Figure 2F), indicating a rapid transition from its cis to trans isomers. In contrast, without SNCs, no significant change was detected after 75 min in the dark (blue line, Figure 2E).

Our experimental evidence points to a fascinating dual interaction mechanism between the azobenzene moieties and the SNCs. In terms of quantum yield,^58^ the optical absorbance of bare and metal-doped PMPs clearly demonstrates that the SNCs enhance the response of the system when irradiated with NIR light. When only SNCs (0% Azo-PMP-1% SNC Supplementary) or only azobenzene (6% Azo-PMP-0% SNC) were present, the system showed no increase in absorbance after irradiation, thus indicating that the key to the transition is the synergy of both. This behavior is interpreted as an isomerization of azobenzene catalyzed by the SNCs in the NIR window, which increases the quantum yield of the system from 0. This catalytic effect is the first interaction between azobenzene and the SNCs. In particular, as explained above, we believe that the SNCs can release photoelectrons that can be captured by the azobenzene moieties, thus populating their LUMO level. The distance between the Fermi level of the SNCs and the LUMO line of azobenzene must be less than about 2 eV, which is the gap between the HOMO and LUMO lines of azobenzene^59^ and can be easily reached by the photoelectrons released from noble metal nanoparticles under resonant excitation.^60^ Photoelectron emission is an ultrafast process that can result in an accumulation of charge^60^ that can be scavenged and transferred through the azobenzene moieties.^61^

The second step observed in our experiments is the so-called dark isomerization effect. It occurs when the wavelength used for excitation is absorbed either by azobenzene or by the SNCs. In particular, in the case of a NIR laser (785 nm), the release of photoelectrons stops when the irradiation ceases and the charge flow reverses as the SNCs facilitate the *cis*–*trans* reisomerization.

### Enhancement of PMP Photoresponse to Coherent and Incoherent Light

**Figure 3:**
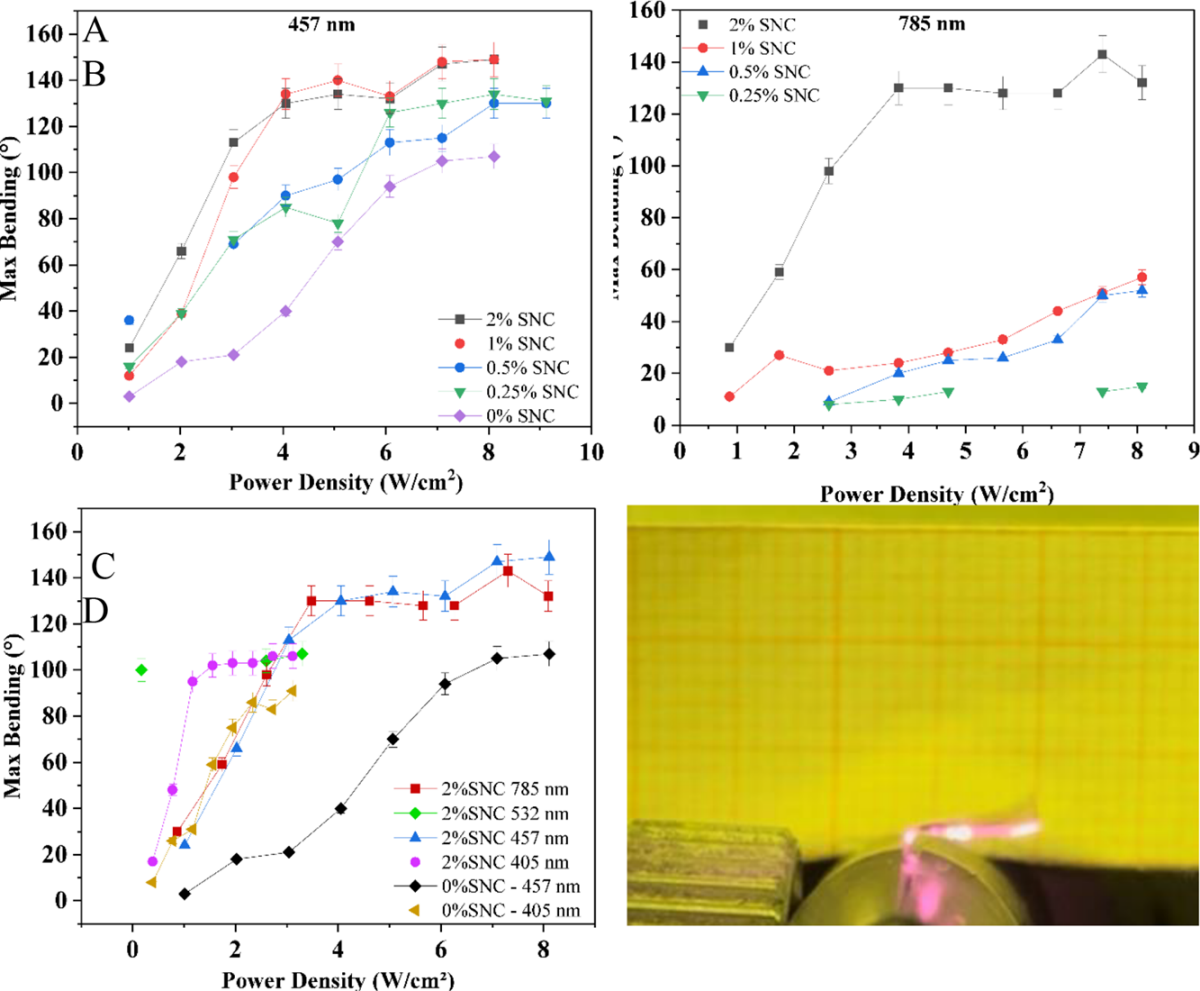
(A) Maximum bending of bare and metal-doped PMPs when irradiated with 457 nm light. (B) Maximum bending of metal-doped PMPs when irradiated with 785 nm light. (C) Maximum bending of 6%azo-PMP-2%SNC and controls irradiated with various wavelengths. (D) 6%azo-PMP-2%SNC vibrating when exposed to NIR light (785 nm). The video is available as Supporting Information.

Our system was tested in a macroscopic environment, with PMPs cut into the shape of cantilevers (5 mm × 1 mm) to see if the photoelectric effect can enhance the bending ability at various wavelengths. To begin with, a laser emitting at a wavelength of 457 nm was tested since we have previously shown^41^ that the LC mixtures used in this work^29,62^ undergo efficient activation under such conditions (Figure 3A).

The samples considered for this experiment were a control named 6%Azo-PMP-0%SNC^29,62^ without any metal doping and 6%azo-PMP containing 0.25, 0.5, 1, and 2% (w/w) SNCs. All samples folded efficiently, even at low power densities. For example, the control bent up to 15 degrees with an optical power density of 2 W/cm2 (Figure 3A). The metal-doped samples even at low SNC concentrations performed better than the control, showing a clear effect of the SNC concentration. We found that the higher the SNC concentration, the greater the strain. Even though the laser wavelength was not in full resonance with the plasmonic band of the SNCs, its presence was found to enhance the performance of the nanocomposite material. This finding may relate to the residual absorbance of the SNCs between 400 and 500 nm (Figure 1C), which may increase upon broadening of their plasmonic spectrum as a result of the onset of particle aggregation.^63^ The best performance was obtained with 6%Azo-PMP-1% and 2%SNC, as both samples showed a strain that was approximately 6-fold higher than the control at 3 W/cm^2^ (Figure 3A). Interestingly, we observed a saturation effect, as there was no further improvement at SNC concentrations above 1% (Figure 3A).

The same experiment was repeated with polarized light from a laser at 785 nm in order to evaluate the response of bare and metal-doped PMPs to NIR light (Figure 3B). Again, a concentration effect was evident when the metal content was increased. The minimum SNC concentration for efficient conversion of NIR light to elastic energy was identified at 1%. The PMP performance improved by a factor around 4 when the metal content increased from 1 to 2%. Furthermore, the sample containing 2% SNCs went into continuous self-motion (Figure 3D), which is another indication that there is more than just a thermoelastic component to the underlying mechanism. In the past, this effect was only observed when azobenzene was stimulated with UV light, and is possible only in the presence of a reversible *trans*–*cis*–*trans* isomerization process.^64^

Subsequently, the 1% was used (Supplementary Figure 3) and 2% SNC samples were chosen for further characterization with other wavelengths (Figure 3C and Video). In particular, lasers at 405, 457, 532, and 785 nm were compared to explore the interactions between azobenzene and the SNCs (Figure 3C). The results confirmed that the SNCs were able to improve the conversion of light to mechanical work at all wavelengths. Notably, the metal-doped samples performed better than the controls, even when the light was optimally or suboptimally absorbed by azobenzene (405 and 457 nm). Under these conditions, 6%Azo-PMP-2%SNC folded 1.5 to 4-fold more than controls (Figure 3C). More interestingly, NIR light was able to trigger the movement of the metal-doped samples as efficiently as at 405 and 457 nm (Figure 3C), whereas the control remained motionless when irradiated at 785 nm. This result shows a clear parallel between the mechanical and optical characterization of the metal-doped samples. Indeed, the behavior described here supports the hypothesis that the NIR light-induced motion is not only due to a thermoelastic effect but also to some interaction between azobenzene and the SNCs. We demonstrated that the SNCs are able to mediate both an increase in their host temperature and a change in the optical absorbance of azobenzene when hit by NIR light (Figure 2). Metal-doped PMPs are able to not only bend (Figure 3) but also self-vibrate (Figure 3D). These dynamics suggest that, when the 785 nm laser is turned on, the SNCs release photoelectrons which excite the azobenzene moieties to isomerize and, when turned off or shadowed, they recover the emitted charge, thus yielding bending and self-vibration.

We correlated all of these data to the spectrum of solar irradiance measured outside the atmosphere at one astronomical unit from the sun in September 2001^65^ (Supplementary Figure 4). All values were then reduced by a constant factor of 50% from 405 to 532 nm and by 5% for 785 nm in order to simulate the effect of atmospheric absorbance. Using these standardized data calibrated with our experimental measurements, we hypothesized quantitatively how the metal-doped PMPs would bend more than bare ones under sunlight exposure. We estimated that with 1% SNCs, the PMP would fold approximately 2.4 times more than the control. When 2% SNCs were added to the mix instead, it responded up to 3.4 times better than the control. These multipliers were calculated considering only the solar energy density at the individual wavelengths tested in the laboratory.

**Figure 4:**
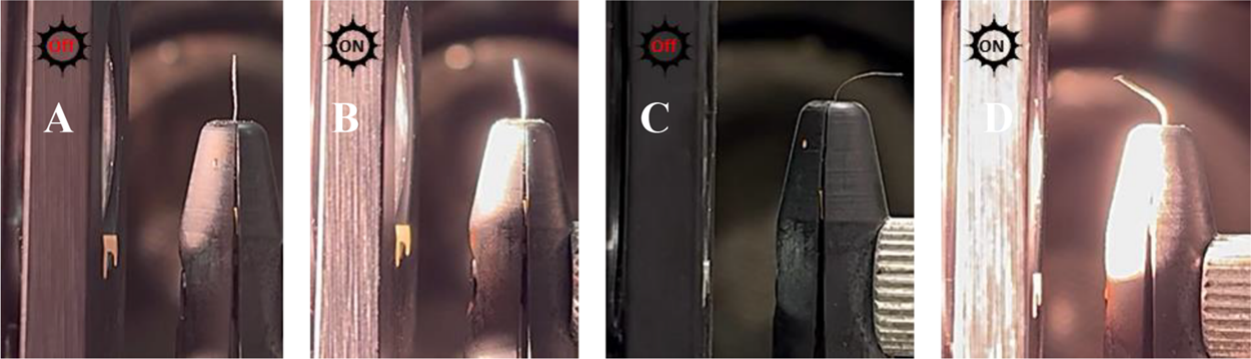
(A, B) Sample 0%Azo-PMP-1%SNC—(A) white light off, (B) white light on, and (C, D) sample 6%Azo-PMP-2%SNC—(C) white light off, (D) white light on.

We also tested our PMPs and SNCs under nonpolarized incoherent light (halogen lamp and natural daylight) to understand if the nanocomposite films can work and thus be suitable for solar energy harvesting under real environmental conditions. Three samples were tested with the halogen lamp, namely, 6%Azo-PMP-2%SNC, 0%Azo-PMP-2%SNC, and 6%Azo-PMP-0%SNC. All samples were stimulated at a high power density of 4100 W/m^2^ showing a broad wavelength range between 400 and 1100 nm. This experiment first shows that no thermoelastic effects are involved in the movement of the metal-doped PMPs and that the use of unpolarized light is only effective for SNC-containing PMPs. In Figure 4A,B, 0%Azo-PMP-2%SNC is shown to bend only 13 degrees, while 6%Azo-PMP-2%SNC responds approximately 10-fold more than the control (Figure 4C,D). This finding confirms our assessment that the thermoelastic effect is only a small fraction of the factors that synergize in the metal-doped PMPs. Furthermore, the increased bending of the PMPs in the presence of azobenzene shows that the SNCs are critical in allowing their isomerization when using nonpolarized white light. When 6%Azo-PMP-0%SNC was tested under unpolarized white light, its deflection was only 9 degrees, thus reconfirming that the control sample needs both polarized and UV-rich light.

In addition, we used a broad-spectrum xenon lamp (312–1800 nm) to evaluate the performance of the metal-doped PMPs under conditions mimicking sunlight exposure. To test our PMP as an engine for a proof-of-concept harvesting system, we coupled a copper laminated PMP with a flexible PZL material. Using two distinct experimental setups, this integration aimed to illustrate the potential of the new materials to respond to sunlight and generate electricity.

In the first experimental setup, we introduced a chopper into our system to periodically dim the light from the xenon lamp and to irradiate the PMP with alternating periods of light. This cyclic on–off exposure triggered a smooth movement within the material, leading to a constant generation of electric potential (Supplementary Figure 5 and Supplementary Videos). The experiments were performed by changing the power density of the radiation. This approach helped us simulate the response of the new material under real-world conditions, where the exposure to sunlight changed during the whole day. The data showed that by increasing the power density, the piezo energy output increased accordingly (Supplementary Figure 5). For the second approach, we implemented what we call the 'pendulum' experiment. In this scenario, the PZL device was subjected to a single, powerful pulse caused by the PMP layer when exposed to continuous irradiation. In this case, using the same PMP–PZL configuration of the first experiment, the PMP is able to forcefully overcome the edge of the PZL device that finally starts to oscillate. This intense one-shot activation aimed to test the ability of this system to generate energy under different conditions and with different approaches (Supplementary Figure 5 and Video).

The combination of both setups provided valuable insights into the dynamic behavior of our metal-doped PMPs under different light exposure conditions. Our tests demonstrated the feasibility of our concept, highlighting its potential for harnessing sunlight to generate electricity. However, there is still a need for optimization to further improve these initial results. For example, in future work, we will try to further adapt the oscilloscope impedance to obtain a higher and clearer voltage signal. Despite these challenges, the results obtained are promising and open the scene for a wide range of applications. The adaptation of these initial results can span various fields, showing the versatility of the SNC-doped PMPs. We predict that the refinement of the experimental procedure will progressively push the limits of its potential applications, thus harnessing the inexhaustible power of the sun to meet the growing demand for energy. Furthermore, alternative approaches are also possible as Park et al. showed. This paper describes a light-transformable and healable triboelectric nanogenerator based on photofluidization of azobenzene polymer, which provides a reversible method for improving and restoring energy harvesting performance in wearable technologies.^66^

**Figure 5:**
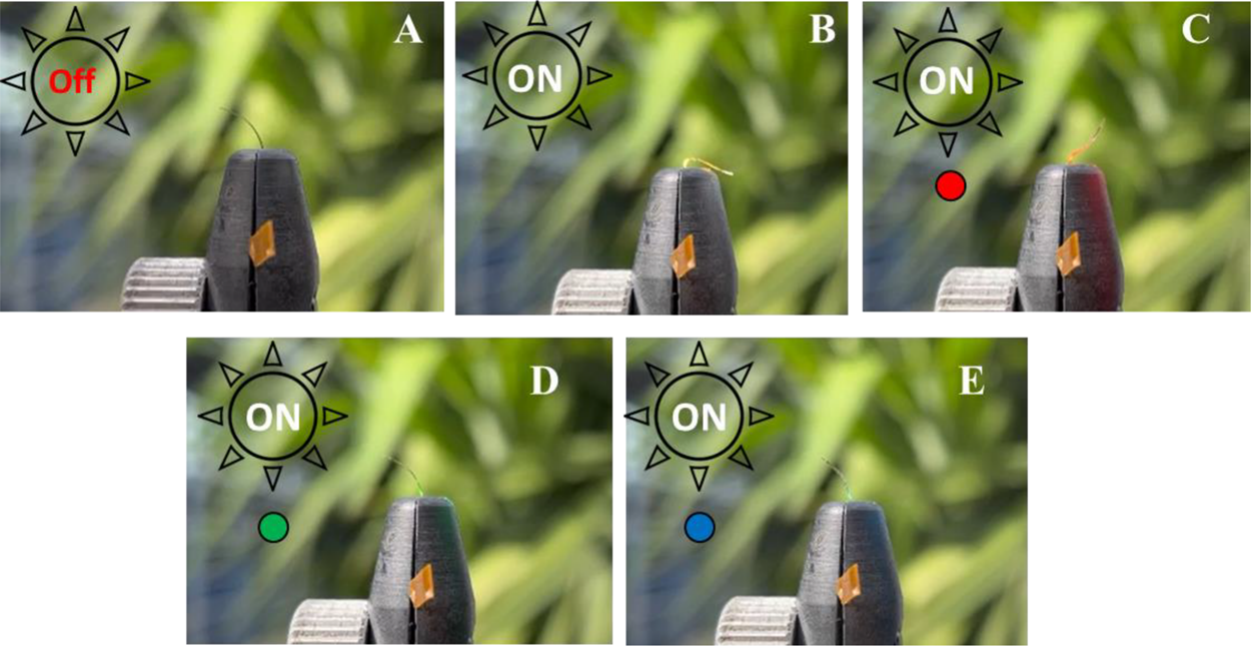
Outdoor experiments under real solar conditions. Unpolarized sunlight irradiation of 6%Azo-PMP-2%SNC—(A) solar light off, (B) solar light on (550 W/m^2^), (C) solar light on with red high-pass filter (350 W/m^2^), (D) 450–600 band filter (45 W/m^2^), and (E) 350–550 band filter (75 W/m^2^).

We also tested the PMPs outdoors using focused, natural, or filtered unpolarized sunlight in an effort to verify the functionality of our material as a tool for solar energy harvesting in a relevant environment. As illustrated in Figure 5, metal-doped PMPs are capable of bending where the control does not. Indeed, the metal-doped PMP is capable of bending and vibrating when irradiated in bright sunlight or even behind a 615 nm high-pass filter for compatibility with different uses of visible light. Instead, the control bends just 1° and remains motionless when visible and UV light is removed (Table 1). Furthermore, the metal-doped PMP readily enters into self-vibration while being irradiated with ambient sunlight.

**Table 1:** Max Bending of Metal-Doped PMPs and Control when Irradiated with Unpolarized Sunlight

name	max bending (deg)
6%Azo-PMP-2%SNC (sunlight)	151
6%Azo-PMP-2%SNC (sunlight + red filter)	86
6%Azo-PMP-0%SNC (sunlight)	1

These results are unprecedented and remarkable for solar energy harvesting for two reasons: (i) the first is the use of ambient sunlight using low-content azobenzene materials and (ii) the second is that the metal-doped PMPs respond efficiently to unpolarized, artificial, or natural light, thereby improving the efficiency and reducing the cost of any device that can use such materials for photomechanical conversion (Figure 5D and Supplementary Video).

## Conclusions

In conclusion, we prepared novel nanocomposite films with a specific LC blend and SNCs tailored to resonate around 785 nm, with the ultimate goal of obtaining light-mobile polymers for efficient solar energy harvesting. We demonstrated that the addition of SNCs to the LC matrix allows for the isomerization of azobenzene as it is irradiated with near-infrared light at 785 nm. We found that the noble metal dopant enhanced the ability of its host to bend when stimulated with all wavelengths we tested in the UV and visible range. We demonstrated that the new materials exhibited an impressive response to unpolarized white light and natural daylight, where the strain observed outdoors in the presence of 2% noble metal was 100 times more than that of the bare polymer. Finally, we provided evidence of the integration of our metal-doped films into novel photovoltaic modules based on PZL polymer coupling. We expect our work to establish a potential cornerstone for the use of PMPs in solar energy harvesting.

## Supporting Information Available

Polarized microscopy photographs, absorbance of PMP with silver nanocuboids in the absence of azobenzene, 1% nanocuboids pmp max bending, solar irradiance measured at one astronomical unit, and proof-of-concept PMP/PZL integration for energy harvesting (PDF)
2% nanocuboids under sunlight (MP4)
Control samples under white light (MOV)
1% nanocuboids under white light (MOV)
2% nanocuboids under sunlight (MP4)
2% nanocuboids irradiated at 784 nm (MOV)
2% nanocuboids under sunlight (MP4)
Control samples under sunlight (MP4)

## Supplementary Material

ot3c00227_si_002.mp4

ot3c00227_si_003.mov

ot3c00227_si_004.mov

ot3c00227_si_005.mp4

ot3c00227_si_006.mov
