# Supplementary material for: Photomobile Polymer–Piezoelectric Composite for Enhanced Actuation and Energy Generation
Source: ACS Appl Opt Mater. 2023 Sep 28;1(10):1651–60. doi: 10.1021/acsaom.3c00227 (PMC10616835; doi:10.1021/acsaom.3c00227)
Supplement: Supplementary file 1 — ot3c00227_si_001.pdf [file ot3c00227_si_001.pdf]

Supporting information

Photomobile Polymer-Piezoelectric Composite for Enhanced Actuation and Energy Generation

Domenico Sagnelli[1], Amalia D'Avino[1], Massimo Rippa[1], Ambra Vestri[1], Valentina Marchesano[1],*

Giuseppe Nenna[2], Fulvia Villani[2], Gustavo Ardila[3], Sonia Centi[4], Fulvio Ratto[4], Lucia Petti[1]

[1]Institute of Applied Sciences and Intelligent Systems of CNR, 80072 Pozzuoli, Italy

[2]ENEA, Italian National Agency for New Technologies, Energy and Sustainable Economic Development, Portici Research Centre, Portici, 80055 Naples, Italy

[3]Univ. Grenoble Alpes, Univ. Savoie Mont Blanc, CNRS, Grenoble INP, IMEP-LaHC, F-38000 Grenoble, [4]Nello Carrara Institute of Applied Physics of CNR, 50019 Sesto Fiorentino, Italy

France

* Correspondence and requests for materials should be addressed to: domenico.sagnelli@isasi.cnr.it

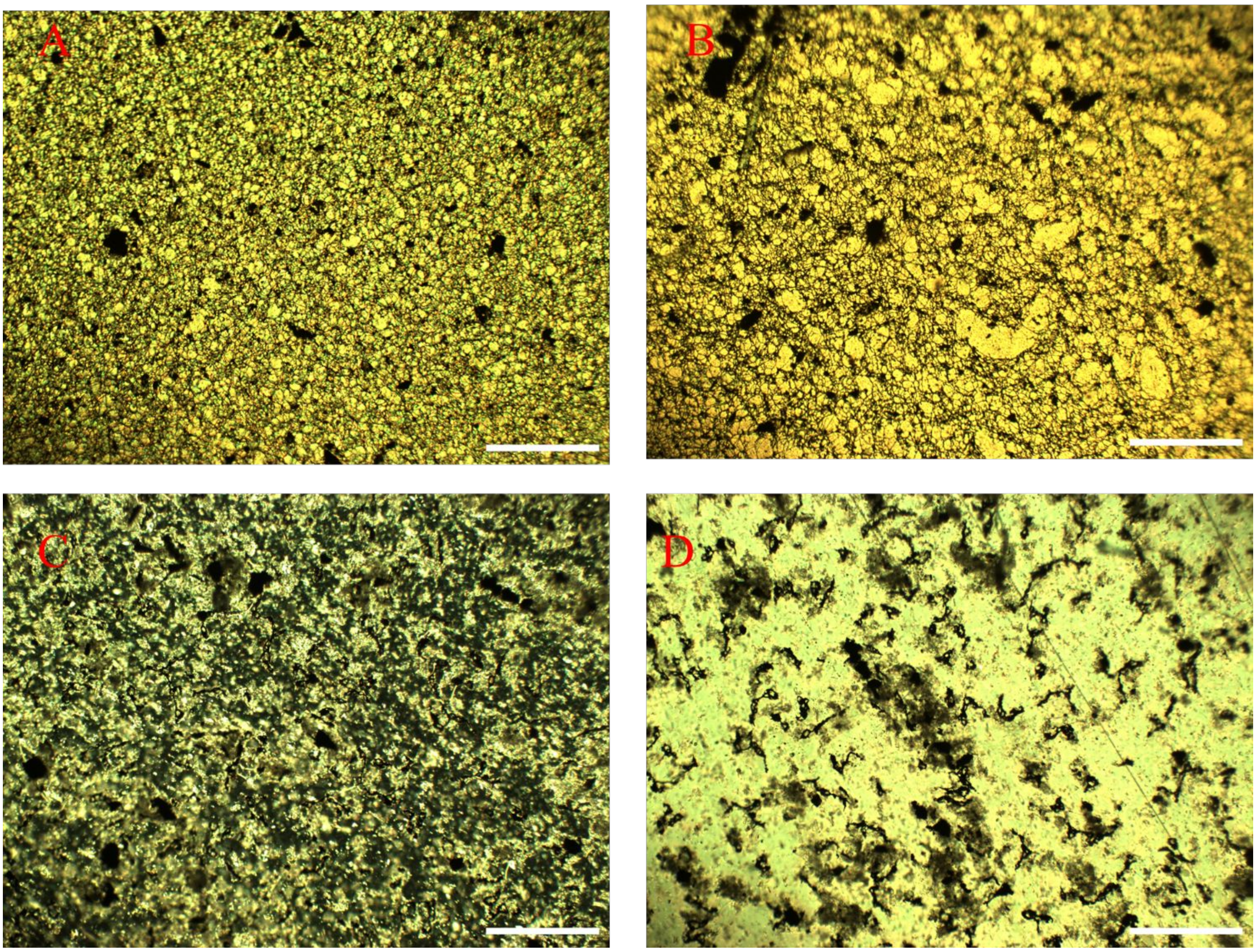

Figure S1. A&B) 6%Azo-PMPs doped with silver nano-cuboids A) PMP with rubbing parallel to the polarizer. B) Rubbing is tilted 45 degrees with respect to the polarizers. C&D) 0%Azo-PMP. C) PMP with rubbing parallel to the polarizer. D) The rubbing is tilted 45 degrees with respect to the polarizers.

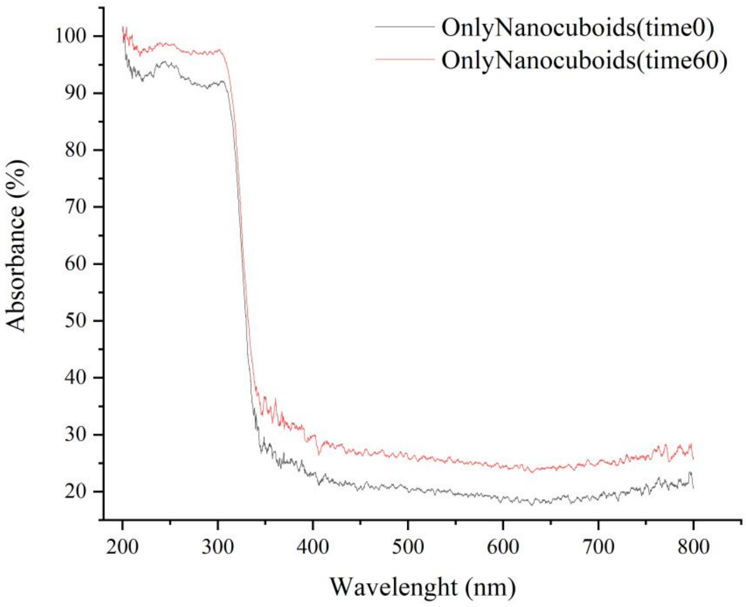

Figure S2. Absorbance of 0% Azo-PMP-SNC, before and after 785nm irradiation. The liquid crystal mixture is the same as the full composite except for the azobenzene.

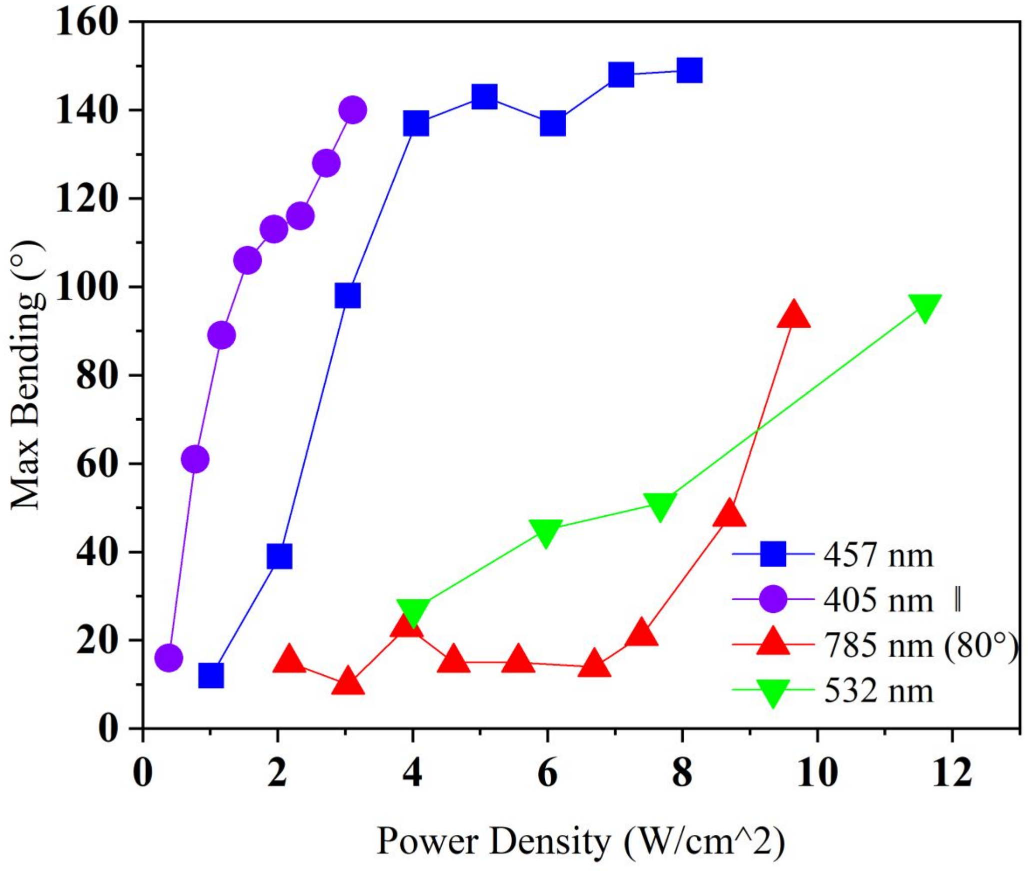

Figure S3. Maximum bending of 6%azo-PMP1%SNC irradiated with various wavelengths.

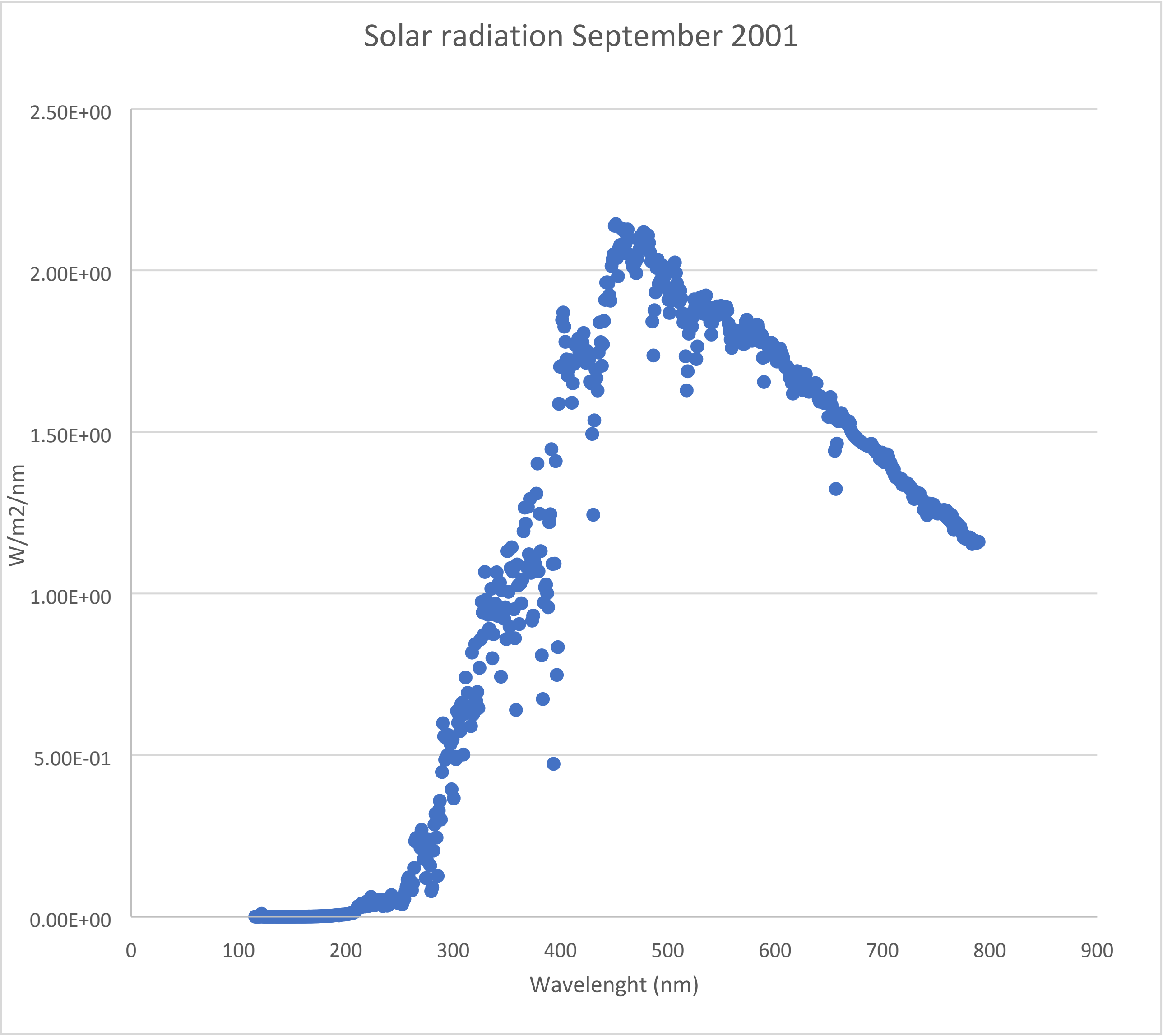

Figure S4. Solar irradiance measured at one astronomical unit outside the atmosphere in September 2011. [1]

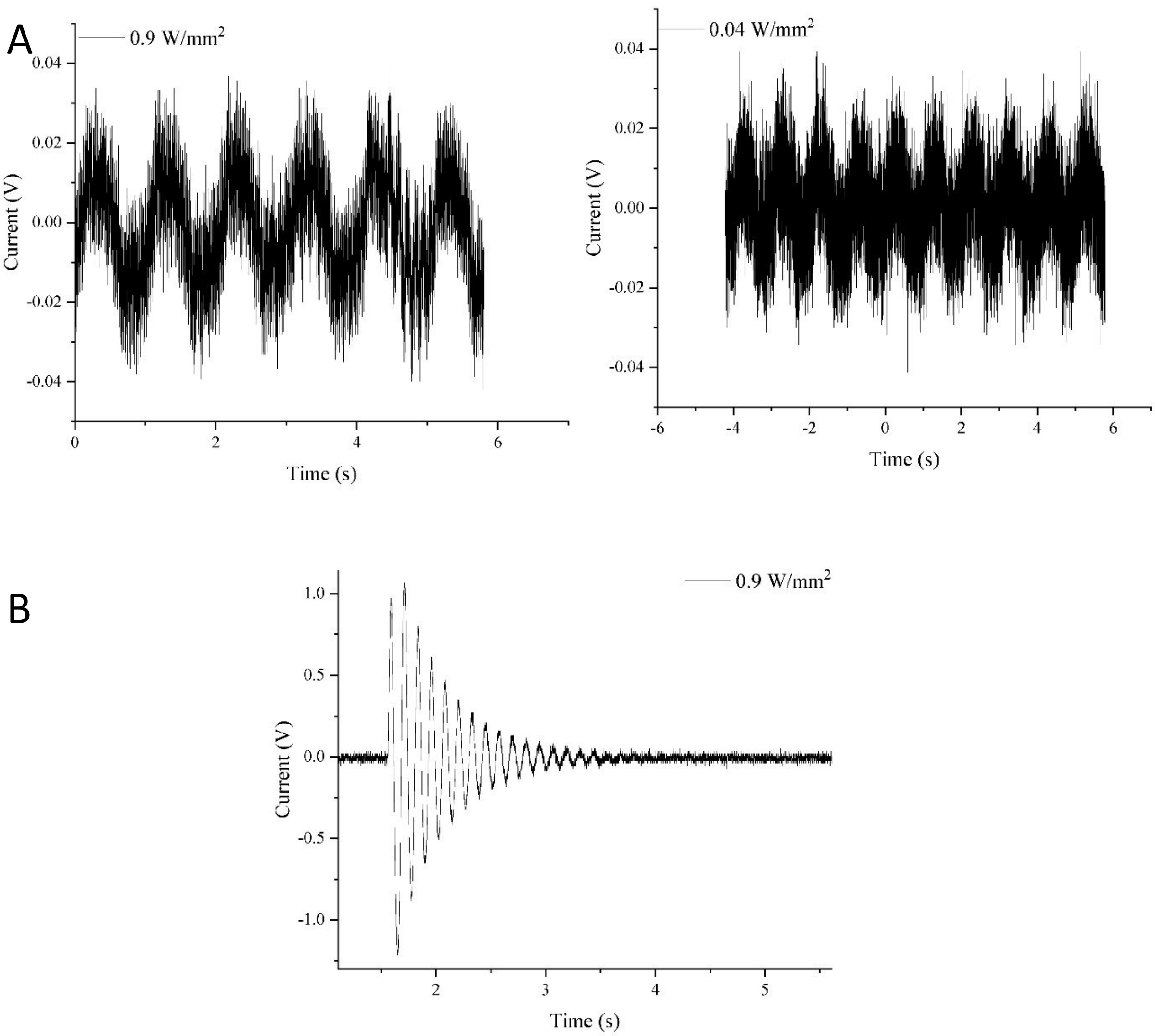

Figure S5. Proof of concept of PMP/PZL integration. A) Cyclic on-off irradiation at 0.9 W/mm^2 and 0.8 W/mm^2 B) The so-called pendulum experiment performed at 0.9 W/mm^2

1. SSI Climate Data Record (CDR) - Reference Spectra Available online: https://www.ncei.noaa.gov/data/solar-spectral-irradiance/access/ancillary-data/tsi-ssi_v02r01_reference-spectra_c20170327.txt.
